# Stability scheme of ZnO-thin film resistive switching memory: influence of defects by controllable oxygen pressure ratio

**DOI:** 10.1186/1556-276X-8-483

**Published:** 2013-11-16

**Authors:** Hsin-Wei Huang, Chen-Fang Kang, Fang-I Lai, Jr-Hau He, Su-Jien Lin, Yu-Lun Chueh

**Affiliations:** 1 Department of Materials Science and Engineering, National Tsing Hua University, Hsinchu 30013, Taiwan; 2 Department of Electrical Engineering and Institute of Photonics and Optoelectronics, National Taiwan University, Taipei 10617, Taiwan; 3 Department of Photonics Engineering, Yuan Ze University, Taoyuan 32003, Taiwan; 4 Center for Nanotechnology, Material Science and Microsystem, National Tsing Hua University, Hsinchu 30013, Taiwan

**Keywords:** ZnO, O_2_ partial pressure, Oxygen defects, Resistive change memory

## Abstract

We report a stability scheme of resistive switching devices based on ZnO films deposited by radio frequency (RF) sputtering process at different oxygen pressure ratios. *I*-*V* measurements and statistical results indicate that the operating stability of ZnO resistive random access memory (ReRAM) devices is highly dependent on oxygen conditions. Data indicates that the ZnO film ReRAM device fabricated at 10% O_2_ pressure ratio exhibits the best performance. Transmission electron microscopy (TEM) and X-ray diffraction (XRD) of ZnO at different O_2_ pressure ratios were investigated to reflect influence of structure to the stable switching behaviors. In addition, PL and XPS results were measured to investigate the different charge states triggered in ZnO by oxygen vacancies, which affect the stability of the switching behavior.

## Background

Recently, resistive random access memory (ReRAM) has intensively attracted much attention, which will become one of the potential candidates in next-generation memory, owing to its advantages, including nonvolatility, high speed, high density, and low power consumption [1,2]. From the materials science point of view, many metal oxide materials, such as perovskite-type oxides, ferroelectric oxides, and binary transition metal oxides, have exhibited differently resistive switching characteristics [3-5]. Up to date, the best switching behaviors of ReRAM devices were observed on the binary transition metal oxides, such as NiO and TiO_2_[6-9].

ZnO is one of binary transition metal oxides with several applications as optoelectronics because of a wide optical direct bandgap of approximately 3.37 eV, a high exciton binding energy of around 60 meV, and has been exhibited excellent resistive behavior [10-12]. However, optimized conduction in ReRAM applications for the ZnO-based ReRAM is not well investigated yet, whose defects resulted from pristine conditions or doping in the ZnO film are not easily controlled. Most of the studies have indicated that migration of oxygen ionic atoms plays an important role in the resistive switching process [13,14]. The conductivity of metal oxide is highly sensitive regardless whether the oxygen atom existed at a lattice site or not.

In this regard, by varying partial pressures of oxygen gases (O_2_) during sputtering process, native defects related to resistive behavior in the ZnO layer, including oxygen vacancies, Zn vacancies, oxygen interstitials, and Zn interstitials, were investigated in detail, respectively [15-17]. The amount of these defects would significantly affect the resistive switching behaviors of the ZnO layers as well as the stability. Here, photoluminescence (PL) and X-ray photoelectron spectroscopy (XPS) were used to identify the native defects.

## Methods

ZnO films of 100-nm thick were deposited on Pt/Ti/SiO_2_/Si substrates at room temperature (RT) by RF sputtering of the ZnO target at different O_2_ pressure ratios from 0%, 10%, 33% to 50%. Pt electrode with a diameter of around 200 μm was used to fabricate a symmetrical metal-insulator-metal (MIM) sandwich structure by shadow mask. The *I*-*V* behaviors of these devices under different temperatures were measured by a Keithley 4200 semiconductor parameter analyzer (Keithley Instruments Inc., Cleveland, OH, USA). The crystalline structures of the ZnO films were examined by X-ray diffraction (XRD) and transmission electron microscopy (TEM). The PL measurements were performed using a He-Cd laser with an excitation wavelength of 325 nm at RT to unveil the defects in the ZnO layer. XPS was used to observe the chemical bonding energy with different oxygen states.

## Results and discussion

**Figure 1:**
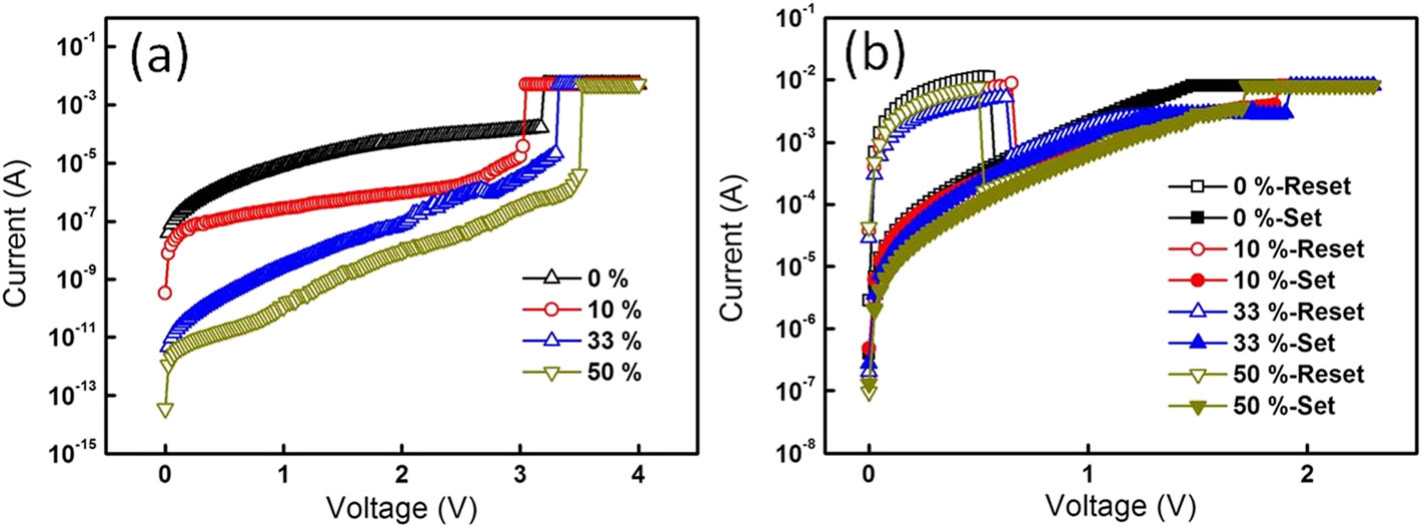
***I*-*V *curves. (a) ***I*-*V* curves at the forming process and **(b)** typical *I*-*V* curves of ZnO ReRAM devices at different O_2_ pressure ratios.

Figure 1a shows the forming process of Pt/ZnO/Pt devices, with which ZnO layers were deposited at different O_2_ pressure ratios from 10%, 33%, and 50%, respectively, while the deposition of ZnO layer at a pure Ar ambient, denoted as 0% O_2_ pressure ratio, acts as the reference for the comparison. Obviously, the initial resistance state increases as oxygen pressure ratio increases. The increasing initial resistance state of the ZnO layer with the increasing O_2_ pressure ratios was contributed from a compensation process of oxygen defects in the ZnO layer. A 'soft breakdown’ process has to be applied at these devices to form a conductive path, in which the current rapidly approaches to the current compliance (8 × 10^-3^ A) at the applied voltage >3 V. This process is called forming process. After the forming process, Pt/ZnO/Pt ReRAM devices could be operated at different resistive status. The corresponding typical *I*-*V* curves at varied O_2_ pressure ratios are shown in Figure 1b. All *I*-*V* curves reveal that the current increases to reach the current compliance as the positive bias was applied (approximately 1.5 to 2 V). The process is called set process, in which the resistive state of the ReRAM device is at the low-resistance state (LRS) due to the formation of conductive filaments in the ZnO layer. Subsequently, a significant current drop, namely reset process, could be achieved as a positive bias of approximately 0.6 V was applied at the same bias polarity. The resistance of the device is thus turned back to the high-resistance state (HRS), in which the conductive filaments are broken by joule heating in a local region triggered by high current flux. The typical switching phenomenon controlled by the same bias polarity is called unipolar switching [18,19].

**Figure 2:**
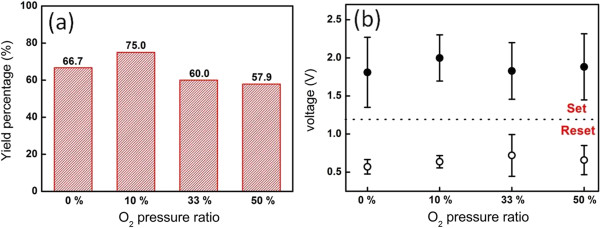
**Yield percentage and set/reset voltage distribution. (a)** Yield percentage of the cells at different O_2_ pressure ratios. **(b)** Distribution of set/reset voltages at different O_2_ pressure ratios.

To shed light on how the stability of the device operates at different O_2_ pressure ratios, statistical results based on yields of switching characteristics were constructed as shown in Figure 2a, in which the statistical results were measured over 20 devices at each O_2_ pressure ratio. The yield is defined as the ratio of the switching devices being successfully operated to 100 cycles. Interestingly, all devices at all O_2_ pressure ratios exhibit yields >50% with successfully operated cycles of >100 cycles. The devices at the 10% O_2_ pressure ratio had the highest yield of approximately 75%, while at O_2_ pressure ratio of around 50%, the yield reduces to 58%. The corresponding statistical results on the deviation distribution of set and reset voltages were shown in Figure 2b. The smallest difference at set/rest voltages could be found at 10% O_2_ pressure ratio, indicating that the ZnO ReRAM devices at the 10% O_2_ pressure ratio can have very stable operation condition compared with that of devices at other O_2_ pressure ratios.

**Figure 3:**
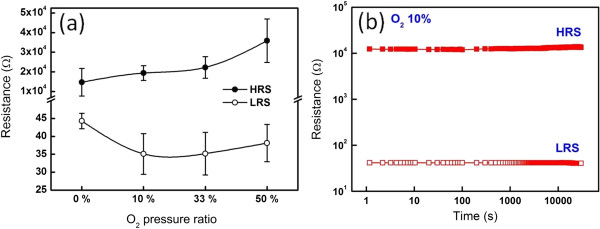
**Resistance ratios and retention performance. (a)** Resistance ratios of HRS/LRS at different O_2_ pressure ratios. **(b)** Retention of a ZnO ReRAM device at the 10% O_2_ pressure ratio.

Figure 3a shows resistive changes of LRS and HRS at different O_2_ pressure ratios, in which the deviation distribution in LRS and HRS was found. The resistance deviation at the LRS for each condition is small (about 40 Ω in average), while the resistance in the high-resistance state increases with increase of O_2_ pressure ratios. Note that the smallest deviation range in the HRS can be achieved at the 10% O_2_ pressure ratio, indicating that injecting of O_2_ molecules during the deposition of the ZnO layer can stabilize the resistive switching behavior. The ratios of HRS/LRS are relatively low at low O_2_ pressure ratios, while the ratio of HRS/LRS increases with the increase of O_2_ partial pressures. The corresponding retention performance at 10% O_2_ pressure ratio was measured as shown in Figure 3b, for which the device can be stably and continuously operated at 30,000 s.

**Figure 4:**
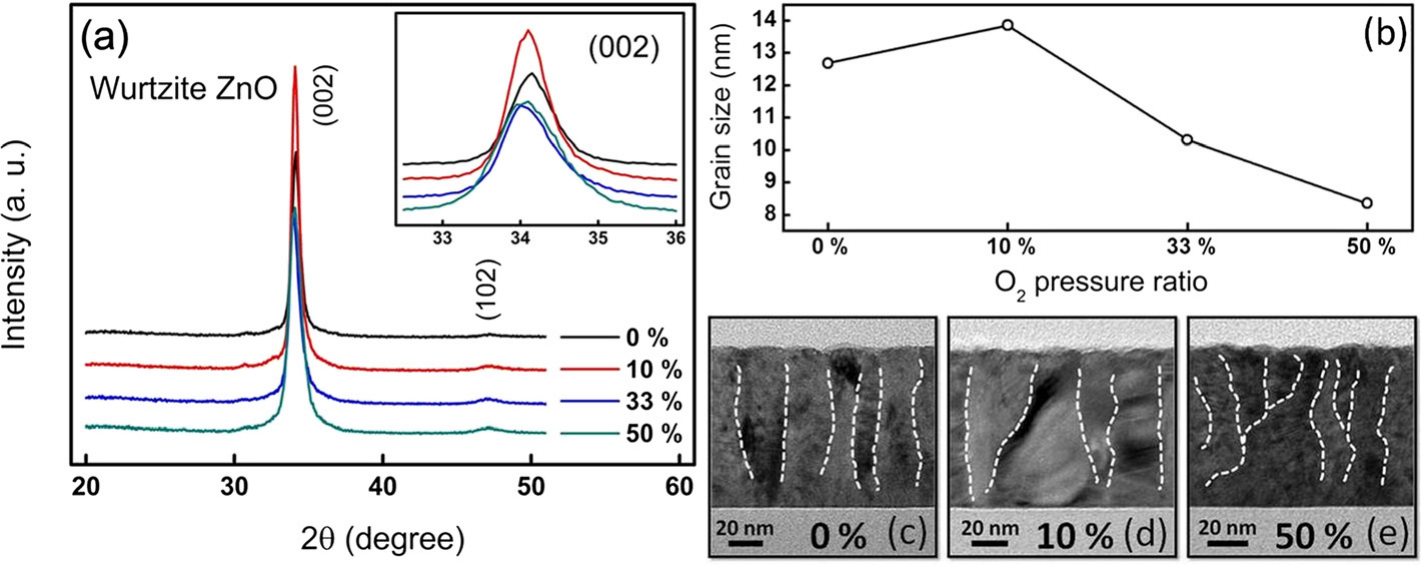
**XRD results and TEM images. (a)** XRD results of ZnO films deposited at different O_2_ pressure ratios. **(b)** Grain size, as the function of varied O_2_ pressure ratios. **(c-e)** Microstructures of ZnO films at different O_2_ pressure ratios.

Based on these statistical results, we consider what role of the oxygen ratios dominates the switching behaviors in the ZnO layer and why the 10% O_2_ pressure ratio can stabilize the ZnO ReRAM device. Many studies have indicated that conductive filaments are generated from the grain boundaries after the forming process [20-22]. The grain boundaries are considered as a defective source because the atoms aligned at these regions are disordered, for which generation of leakage paths is considered as the result of the defects along grain boundaries triggered by electric field. To reveal the grain boundaries, the grain sizes of the ZnO film at different O_2_ pressure ratios are imperative, with which the XRD spectra were measured as shown in Figure 4a. The magnified (002) peaks were shown in the inset. A small shift due to a lattice expansion that resulted from the movement of oxygen ion into the ZnO lattice can be observed when the O_2_ partial pressure increased. A (002) preferred orientation can be indexed as the reference to calculate the grain sizes as the function of O_2_ pressure ratios, using Scherrer equation given by

(1) $$D=\frac{0.89\lambda }{\beta \;\text{cos}\;\theta },$$

where *D* is the grain size, *λ* is the characteristic wavelength of CuK_α_ radiation, *β* is the full width at half maximum (FWHM) of the diffraction peaks, and *θ* is the reflective angle [23]. The calculated grain size dispersion as the function of O_2_ pressure ratios is plotted in Figure 4b. Obviously, the grain size of the ZnO layer increases with the increase of the O_2_ pressure ratio, with which the largest grain size of approximately 13.6 nm can be achieved, while it decreases to about 8 nm after O_2_ pressure ratio increases to 50%. Figure 4c,d,e shows the corresponding TEM images of ZnO films deposited at pure Ar (0% O_2_), 10% O_2_, and 50% O_2_ pressure ratios, respectively. The largest grain size for the ZnO layer can be observed at the 10% O_2_ pressure ratio, while the grain size of the ZnO layer decreases after O_2_ pressure ratio reaches to 50%. The findings are consistent with the XRD results and are similar to reports from Meng and dos Santos [24] and Kong et al. [25]. From the distribution of grain size, the grain size increases slightly at lower O_2_ pressure ratios (<10%). It is generally believed that the formation of grain agglomerations in films deposited under the introduction of oxygen gas might be due to the high-energy neutral oxygen atoms. The high-energy neutral oxygen atoms might accelerate the grain growth [26]. As a result, the grain size decreases when the oxygen partial pressure increased above 33% [27,28]. Typically, a small grain structure usually provides many multiple conducting paths, resulting in an unstable operation [20]. This is why the very stable switching behavior (the highest yield) can be achieved at the 10% O_2_ pressure ratio with the smallest grain boundary volume, with which the largest grain size can be accompanied.

**Figure 5:**
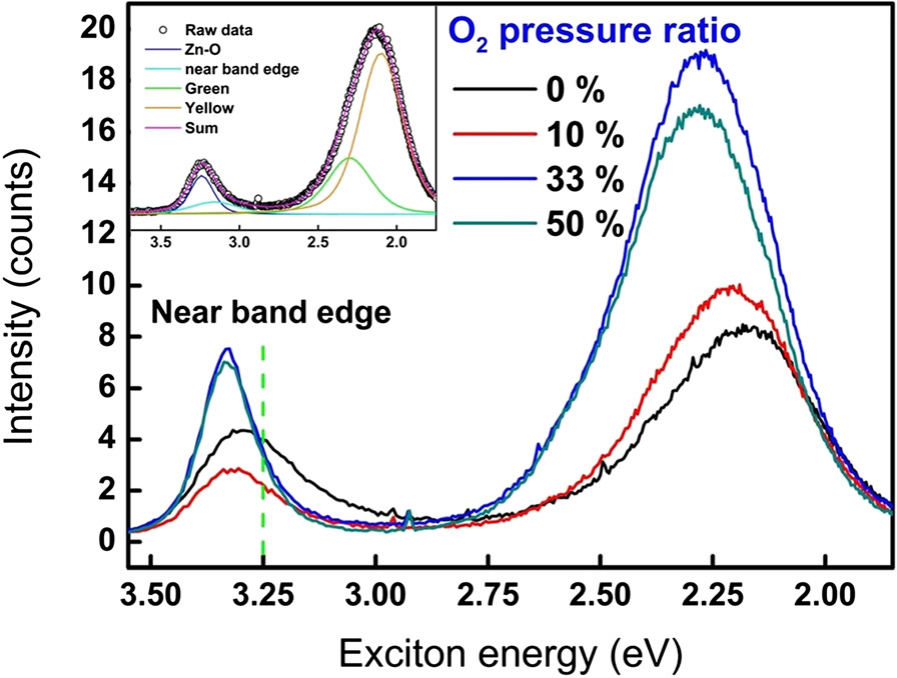
**PL spectra of ZnO films with varied O**_**2 **_**pressure ratios.**

On the other hand, it is generally agreed that the conductive mechanism of the ZnO switching layer is attributed to the migration of oxygen vacancies because the formation energy of the oxygen vacancies is relatively lower than that of other intrinsic defects [17,29-31]. The migration of oxygen vacancies typically results in the generation of a conductive filament during the forming and set processes, which is the conducting mechanism at LRS, namely, a metal-like behavior. However, the species and concentration of the intrinsic defects in ZnO are usually correlated to initial preparation conditions. To shed light on intrinsic defects, PL was measured as shown in Figure 5 at different O_2_ pressure ratios. Two significant peaks at 3.3 and 2.2 eV were obtained, which are ascribed to the direct band edge and green-yellow emissions, respectively. Note that both peaks clearly shift from low energy to high energy as the O_2_ partial pressure ratio increases. From the direct band edge emission, the shift to high energy resulted from the decreasing amount of defects. As a result, introduction of oxygen concentration during deposition could reduce the amount of oxygen vacancies in the ZnO film. In addition, a significant peak can be observed around the direct band edge emission for the ZnO layer at the lower O_2_ pressure ratio, which is called 'near-band edge emission’. Ong and Du reported that the emission of 3.25 eV (near-band edge emission) is caused by a high concentration of defects in the ZnO films [32]. Kim et al. also reported that the emission of 3.23 eV disappeared at the high-pressure oxygen treatment, indicating decreasing of the oxygen vacancies [33]. These reports indicated that the existence of near-band edge emission is due to the oxygen vacancies in the ZnO films. The green-yellow emission at approximately 2.25 eV is directly attributed to oxygen defects. The inset shows a detailed fitting of the PL result, distinctively indicating that the energy shifts to higher energy state resulted from the decrease of the near-band edge emission and the yellow emission triggered by oxygen defects [34]. In addition, the intensity ratio of the defective peak signal/total signal (defect level and band edge) was calculated in order to understand the defect concentration in the ZnO; the intensity ratios are listed in Table 1. As a result, the ZnO film deposited at 10% O_2_ partial pressure exhibits the highest defect concentration compared to the other conditions.

**Table 1:** The intensity ratio from the PL spectra

	**O**_**2**_**ambient (%)**			
	**0**	**10**	**33**	**50**
Intensity ratio	0.66	0.79	0.71	0.72

**Figure 6:**
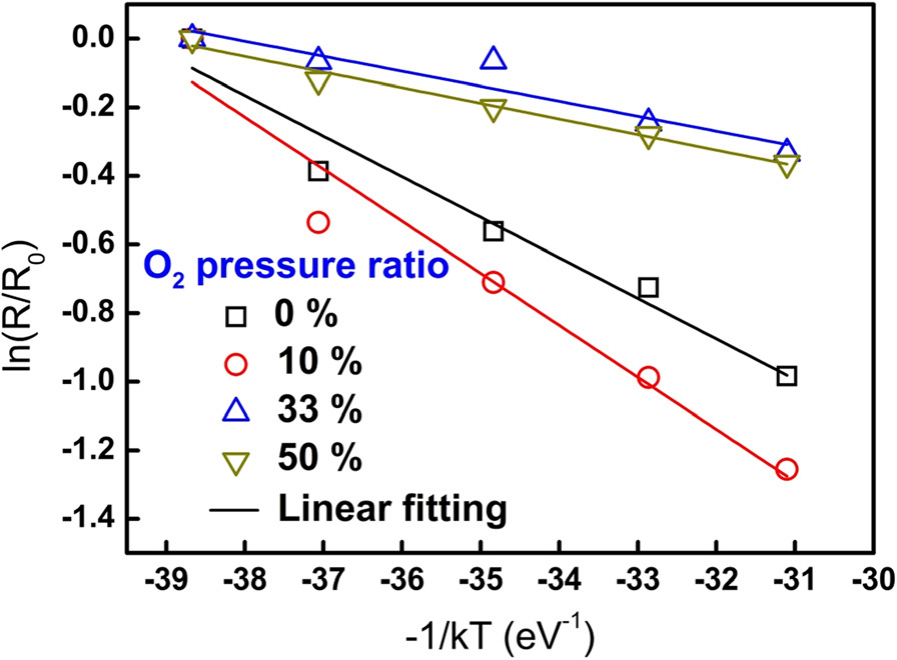
**Plots of ln(R/R**_**0**_**) vs (1/kT) using the Arrhenius equation at different O**_**2**_**pressure ratios.** The slope of each curve represents the activation energy.

An analysis of temperature dependence on resistivity variation was conducted to identify the activation energy of internal defects at different O_2_ pressure ratios. Before the temperature dependence was measured, each cell was operated at five cycles and set at the HRS, in which the resistance was read at 0.1 V. The temperatures varied from RT to 100°C, and the activation energy was calculated using the Arrhenius equation given by

(2) $$R=R_{o}\text{exp}\left(-E_{a}/\mathrm{kT}\right),$$

where *R* is the resistance at different temperatures, *R*_o_ is the resistance at 300 K, *E*_a_ is the activation energy for electrons migrating in the ZnO layer in the HRS, *k* is the Boltzmann constant, and *T* is the temperature which varied from 300 to 373 K [35]. The linear fitting which resulted from the Arrhenius equation was shown in Figure 6, and the distribution of the activation energies can be extracted to be 0.118 to 0.046 eV at different O_2_ pressure ratios as shown in Table 2. Consequently, *E*_a_ located at approximately 0.15 and around 0.05 eV under the conduction band edge can be figured out according to PL and activation energy results caused by oxygen defects in the ZnO film. However, these vacancies exhibit two energy states due to the different charge states, in which the first *E*_a_ (0.15 eV) might be caused by the oxygen vacancy with a positive charge state, $V_{o}^{'}$, and the second one (0.05 eV) is a consequent to the neutral oxygen vacancy, $V_{o}^{0}$[36]. Similar behaviors were also found in TiO_2_ layer by Gu, in which neutral oxygen vacancies form as Ti-O bonding is broken at the set process, resulting in valence electrons surrounding the Ti atoms via the transformation of $V_{o}^{''}$ to $V_{o}^{0}$ confirmed by the first principle calculation [37]. The activation energy shows two values, implying that the valence states of oxygen vacancies might be transformed from $V_{o}^{''}$ to $V_{o}^{'}$ and $V_{o}^{0}$ as the applied voltage increases. Once a higher bias during the forming process was applied, the valence states of the high concentration of neutral *V*_o_ vacancies change, resulting in the formation of high concentration of neighboring Zn ions. As a result, the high concentration of the neighboring Zn ions can be considered as leakage paths, resulting in unstable operation at the higher O_2_ ratio ambient. The leakage paths mean that the filament can be formed and ruptured along these possible paths after every set process. It means that the filaments are not formed and ruptured along the same conductive path, which explains why the unstable *I*-*V* behavior can be observed at the higher O_2_ ratio ambient.

**Table 2:** Activation energy obtained from fitting of Arrhenius relations

	**O**_**2**_**ambient (%)**			
	**0**	**10**	**33**	**50**
Activation energy (eV)	0.118	0.152	0.044	0.046

**Figure 7:**
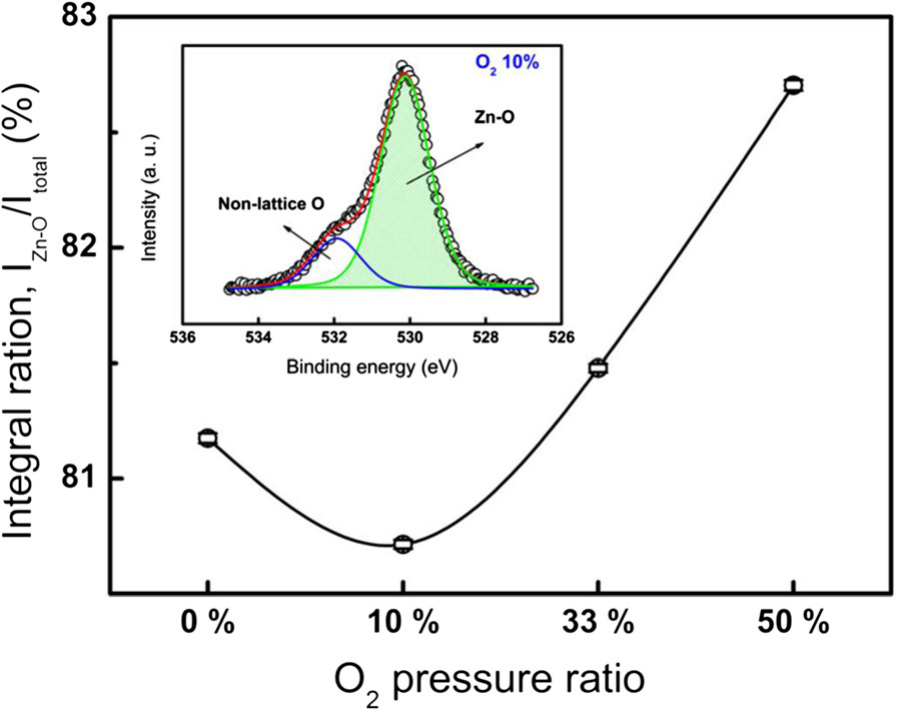
**Integral ratios of Zn-O bondings were calculated from the oxygen signals of the XPS spectra.** The inset shows the fitting result from the 10% O_2_ pressure ratio.

Finally, we try to discuss why different valence states of the oxygen vacancies can be generated during the set process in various O_2_ pressure ratios. PL results implied that the amount of defects differs from various O_2_ pressure ratios which highly depend whether the near-band edge emission exists or not. Therefore, we consider that the amount of oxygen defects plays an important role in the pristine ZnO films. XPS results provide the bonding information of Zn and O, with which fitting results relative to the Zn-O bonding information were conducted as shown in Figure 7. The higher intensity ratio of the *I*_Zn-O_/*I*_total_ indicates that the Zn-O bonding in the higher O_2_ pressure ratio is stronger than that in the lower O_2_ pressure ratio. The results are consistent with the ratios of PL intensities as the function of O_2_ pressure ratios in Table 1. In other words, many defects that existed around the Zn-O bonding in the lower O_2_ pressure ratio might provide some carriers to redox the Zn and O ions under an applied electric field, resulting in different valence states on Zn and O ions. Therefore, the region near the Zn ions may have a relatively higher conductive characteristic with a higher O_2_ pressure ratio, creating more leakage paths, which is a reason of unstable *I*-*V* behavior, because the electrons may migrate along the different paths during itinerant operation. In contrast, the electrons can be trapped by oxygen vacancies at the positive valence state in the lower O_2_ pressure ratio, which might be an explanation for enhancing the stability of ReRAM devices. On the other hand, the results of XRD and PL show a less difference in the lower O_2_ partial pressure (0% and 10%), while they exhibit significant difference as the O_2_ partial pressure increased to 33%. We consider that the lower yield may be due to the failure from leakage paths since it is difficult to keep in high/low resistance state. The 10% case should be the optimized results of controlling the influenced factors.

## Conclusions

In conclusion, the ZnO films prepared by sputtering processes for ReRAM device at different oxygen pressure ratios were measured and investigated. The statistical results from the electrical properties indicate that the ZnO ReRAM devices fabricated at the 10% O_2_ pressure ratio exhibit a very stable *I*-*V* behavior with a high operation yield of approximately 75% compared to the other conditions. The XRD and TEM images showed a large grain aligned at (001) direction in the 10% O_2_ pressure ratio, which limits the electrons migrating along these grain boundaries, thereby stabilizing the switching behavior. From the PL results, a near-band edge emission was observed in the lower O_2_ pressure ratio, owing to the defects that existed in the ZnO film. Two charge states of oxygen vacancies were found according to the calculation using Arrhenius equation, which affect the stability of the switching behavior. Finally, the region near the Zn ions containing a relatively higher conductive characteristic with a higher O_2_ pressure ratio is the reason of unstable *I*-*V* behavior, owing to the creation of more leakage paths, while the electrons trapped by oxygen vacancies at the positive valence state in the lower O_2_ pressure ratio can be an explanation for enhancing the stability of ReRAM devices.

## Abbreviations

FWHM: Full width at half maximum; LRS: Low-resistance state; HRS: High-resistance state; MIM: Metal-insulator-metal; PL: Photoluminescence; ReRAM: Resistive random access memory; RF: Radio frequency; RT: Room temperature; TE: Transmission electron microscopy; XRD: X-ray diffraction; XPS: X-ray photoelectron spectroscopy.

## Competing interests

The authors declare that they have no competing interests.

## Authors’ contributions

HHW carried out the device fabrication and drafted the manuscript. KCF participated in the design of the study. LFI carried out the PL analysis. HJH, LSJ, and CYL carried out the TEM analysis, conceived the study, and organized final version of the paper. All authors read and approved the final manuscript.
